# Optimalisation of the Activation Medium and Effect of Inhibiting Activities of Acid Phosphatase, Lactate Dehydrogenase and β-N-Acetylglucosaminidase on the Fertilisation Success of Eurasian Perch (*Perca fluviatilis* L.)

**DOI:** 10.3390/ani12030307

**Published:** 2022-01-27

**Authors:** Beata Sarosiek, Katarzyna Dryl, Radosław K. Kowalski, Katarzyna Palińska-Żarska, Daniel Żarski

**Affiliations:** 1 Department of Gamete and Embryo Biology, Institute of Animal Reproduction and Food Research, Polish Academy of Sciences, Tuwima Str. 10, 10-748 Olsztyn, Poland; k.dryl@pan.olsztyn.pl (K.D.); r.kowalski@pan.olsztyn.pl (R.K.K.); d.zarski@pan.olsztyn.pl (D.Ż.); 2 Department of Ichthyology, Hydrobiology and Aquatic Ecology, The Stanislaw Sakowicz Inland Fisheries Institute, 10-719 Olsztyn, Poland; k.palinska-zarska@infish.com.pl

**Keywords:** perch, fertilisation, activation solution, acid phosphatase, lactate dehydrogenase, β-N-acetylglucosaminidase, motility

## Abstract

**Simple Summary:**

The purpose of this study is to determine the composition of the optimal activating solution for activating sperm motility and perch eggs, because scientific studies and reproductive procedures should be conducted in stable and repeatable conditions. We found that the best activating solution was composed of 80 mM NaCl, 20 mM KCl, 10 mM Tris, with pH 8.0 and 206 mOsm/kg. In spite of this, we also checked the influence of adding the enzyme inhibitors, such as ammonium molybdate–acid phosphatase inhibitor, gossypol – lactate dehydrogenase inhibitor and acetamide–β-N-acetylglucosaminidase inhibitor on perch sperm motility and successful perch fertilisation. We showed that the addition of acid phosphatase inhibitor in perch semen did not affect the process of fertilisation; in contrast, the addition of lactate dehydrogenase and β-N-acetylglucosaminidase inhibitors significantly decreased the percentage of perch fertilised eggs.

**Abstract:**

Although methods for the artificial reproduction of perch have been developed, a lack of information remains regarding the enzymes present in its semen, as well as their role in the fertilisation process. In this study, we first select the optimal activating solution for perch fertilisation and then determine the inhibition effect of enzymes that have already been reported as present in the sperm of teleosts—acid phosphatase (AcP), lactic dehydrogenase (LDH) and β-N-acetylglucosaminidase (β-NAGase)—on the percentage of motile spermatozoa and fertilised eggs. Of the 8 studied activation media, a solution composed of 80 mM NaCl, 20 mM KCl, 10 mM Tris, with pH 8.0 and 206 mOsm/kg proved to be optimal for perch gametes. The addition of ammonium molybdate (AcP inhibitor) caused no significant reduction in the percentage of fertilised eggs. On the other hand, the addition of 0.25 mM gossypol (LDH inhibitor) and 0.125 M acetamide (β-N-acetylglucosaminidase inhibitor) significantly decreased the fertilisation percentage to 41.1% and 52.4%, respectively, in contrast to the control (89.9 %). Both LDH and β-NAGase thus seem to play a very important role in the perch fertilisation process.

## 1. Introduction

Eurasian perch (*Perca fluviatilis* L.) is a fish species that is highly popular among both producers and consumers. For years, investigations explored the optimisation of its reproduction [1], and many of the resulting optimisation procedures have been successfully applied in aquaculture. Among others, they include protocols for the determination of the maturation stage of females [1], the hormonal stimulation of ovulation, egg quality assessment [2] and in vitro fertilisation [2]. In spite of the immense interest and ample research conducted to date, many aspects of the reproductive biology of perch remain inchoate and require further studies that would facilitate the standardisation of production technology, which is directly dependent on the effectiveness of controlled reproduction [3,4].

Some studies explored the effectiveness of perch fertilisation in various activation media [2]. However, our preliminary experiments with the enzyme inhibitors used in the fertilisation process of perch demonstrated that the percentage of fertilised eggs is often higher in hatchery water (control group) than in the applied medium containing tested inhibitors. Indeed, the addition of some inhibitors caused the pH to decrease, rendering it impossible to conduct the experiment with hatchery water as a positive control of fertilisation. Considering the previous information, we decided to undertake a study with the aim of determining fertilisation success using eight activation media appropriate for perch fertilisation, to ensure a high fertilisation percentage compared to hatchery water (control) and unchanged pH following the addition of inhibitors to these media.

Fish sperm parameters are highly variable in terms of quality. The most important are the volume of ejaculate, morphology, concentration, sperm motility and survival and seminal plasma biochemical composition. Although data regarding the first four parameters may be found in the literature, information about the enzymes present in perch semen is sparse. Although enzymes, such as acid phosphatase (AcP) and lactic dehydrogenase (LDH), have been detected in fish semen for some time [5], the significance of their role in fish fertilisation is still unclear. For example, acid phosphatase present in mammalian sperm takes part in processes, such as sperm hyperactivation, the acrosome reaction and gametes binding [6]. Lahnsteiner et al. [7] observed the negative correlation between LDH activity in rainbow trout seminal plasma and the percentage of achieved offspring. This could indicate sperm damage and the leakage of enzymes into the plasma. Early studies demonstrated the key role of β-N-acetylglucosaminidase (β-NAGase), an enzyme closely connected to acrosome in the semen of trout and ide whose sperm is devoid of it. β-NAGase presence in trout and ide turned out to be more significant than its role in the semen of sturgeon (sperm with the acrosome) [8,9]. The above facts prompted us to establish whether and how the inhibition of AcP, LDH, and β-NAGase activities would affect the fertilisation process of perch, which is a member of the Percidae family, taxonomically distant from the Acipenseridae and Salmonidae.

The purposes of this study is to determine the effects of the inhibition of AcP, LDH and β-NAGase on perch sperm motility and on the success of oocyte fertilisation. In addition, we aim to determine the optimal activating solution composition for activating sperm motility and perch eggs.

## 2. Materials and Methods

### 2.1. Fish Origin and Broodstock Management

The fish used in the experiment originated from the “Żurawia” Fish Farm (Biała Rawska, central Poland). Having been caught from ground ponds in 2014, the fish were transported in bags with oxygen to laboratories at Warmia and Mazury University in Olsztyn (north-east Poland), where they were placed in 300 L pools with a controlled temperature (12 °C) and 14 h photo period (14 L:10 D). A total of 5 days before the semen collection, the males were stimulated with human chorionic gonadotropin (hCG, Argent, Redmont, WA, USA) in a dose of 500 IU/kg.; the female perch (average body weight of 550 ± 27 g) were subjected to the procedure of controlled reproduction described by Żarski et al. [4]. In brief, the fish were subjected to procedures of female maturation stage assessment (after Żarski et al. [1]), and then to hormonal stimulation with the salmon analogue of gonadoliberin (sGnRHa, Syndel, Nanaimo, BC, Canada) in a dose of 25 µg/kg. The collected eggs underwent a quality pre-evaluation (as described by Żarski et al.) [2,4]. Each female, at the time of injection, was scored for its maturity stage. To this end, from each female, an oocyte sample (30–50 oocytes) was taken in vivo using a catheter. Next, the sample was submerged in the cytoplasm clarifying solution (ethanol, 38% formaldehyde and glacial acetic acid mixed at volumetric ratios of 6:3:1), and after 5 min the oocytes were examined under the stereoscopic microscope (Leica MZ12.5, Wetzlar, Germany). Each sample was assigned to 1 of the 6 categories of the oocyte maturation stage (according to Żarski [1]):

Stage I The germinal vesicle (GV) was situated in the oocyte centre, oil droplets were poorly visible;
Stage II Beginning of GV migration, beginning of coagulation of oil droplets, which were clearly visible;
Stage III Migrating GV (above half of the oocyte diameter), oil droplets were clearly visible;
Stage IV The GV was at the oocyte periphery, a large forming oil droplet was clearly visible (the droplet diameter was greater than the GV diameter and it reached the size of about 1/3 of the oocyte diameter) with visible smaller droplets;
Stage V The GV was situated at the oocyte edge, one large (size of about half the oocyte diameter) oil droplet was clearly visible;
Stage VI Oocyte samples taken for analysis were macroscopically transparent; there was no visible GV after they were placed in Serra’s solution (following GVBD), oocytes at the preovulation stage.

For the experiment fish assigned to maturation stage IV and higher, as it was shown by Żarski et al. [1], the eggs obtained from such females were usually of the highest quality.

Briefly, the eggs were checked for the presence of fragmented lipid droplets, being a marker of lowered egg quality [10]. Next, the eggs were checked for the occurrence of any internal damage and/or improprieties, as described by Żarski [4]. Those that revealed no symptoms of deteriorated quality were selected for further procedures. Prior to each manipulation, the fish were anesthetised in MS-222 (Argent, Redmont, WA, USA) solution (150 mg/L).

### 2.2. Determination of Optimal Activating Solution for Perch Gametes

This experiment was divided into two separate experiments, which served to optimise the perch fertilisation rate as required in this study. These aimed to eliminate any variables resulting from the potential interaction of enzyme inhibitors with components of the activation media. In other words, our goal was to make sure that both the semen and the eggs would exhibit full fertilisation capability, and that one of the activation solutions used in the study would be optimal for the perch gametes.

#### 2.2.1. Determination of the Optimal Activating Solution for the Perch Sperm Motility Parameters

The semen of 4 males was used. The experiment served to establish the optimal medium from the solutions analysed for perch sperm motility activation. The following solutions were tested: K—control; hatchery water (ca. 4 mOsm/kg); A—„Lahnsteiner” solution [5] (100 mM NaCl (Sigma-Aldrich, Burlington, MA, USA.), 10 mM Tris (Sigma-Aldrich), pH 7.0, 199 mOsm/kg; B—„Lahnsteiner” solution, pH 8.0, 198 mOsm/kg; C—„Lahnsteiner” solution, pH 9.0, 199 mOsm/kg; D—10 mM NaCl, 10 mM Tris, pH 8.5, 35 mOsm/kg; E—20 mM Tris, 40 mM NaHCO_3_ (Sigma-Aldrich), pH 8.5, 101 mOsm/kg; F—„Woynarovich” solution [11], 0.3% urea (Sigma-Aldrich), 0.4% NaCl, 181 mOsm/kg; G—10 mM HEPES (Sigma-Aldrich), 100 mM NaCl, pH 8.0, 204 mOsm/kg; and H—80 mM NaCl, 20 mM KCl (Sigma-Aldrich), 10 mM Tris, pH 8.0, 206 mOsm/kg (Table 1). Following the addition of 0.5% of albumin (Sigma-Aldrich), the prepared solutions were determined for sperm motility. The activation was conducted in a small Eppendorf tube, where the sperm samples were mixed with activation solutions. A total of 0.5 µL of fresh sperm samples were mixed with 100 µL of the activation solution. Following activation, 1 µL of each sample was transferred to a twelve-well Teflon-coated slide glass (Tekdon Inc. 40521 State Road 64 Myakka City, FL 34251, USA). Motility analysis was carried out using computer-assisted semen analysis (CASA) with Crismas software (Image House CRISMAS Company Ltd. Copenhagen, Denmark). The sperm movement was documented 6 s after activation with a Basler 202 K digital camera (47 frames per s) integrated with an Olympus BX51 microscope. The following sperm motility parameters were analysed: VCL (curvilinear velocity of sperm; μm s^−1^), VSL (straight-line velocity; μm s^−1^), MOT (percent of motile sperm; %) and PRG (percentage of sperm with progressive movements; %).

#### 2.2.2. Determination of the Optimal Activating Solution for Perch Fertilisation

All solutions tested above (Table 1) were used to determine their effects on perch egg fertilisation. The fertilisation was performed following the procedure described by Żarski et al. [2], wherein each egg sample (ca. 100 eggs each) was first activated on a Petri dish in 5 mL of an activating buffer and then after 15 s, 20 µL of a semen mixture (from 3 males, average sperm concentration was 20 × 10^9^ mL^−1^) was added to the sample. After 3 min, each dish was rinsed, first with the respective activating medium to wash out residues spermatozoa, and then with hatchery water. Afterwards, the samples were incubated at 14 °C, and 72 h later the embryonic survival rate was determined according to the following formula: eggs that were fertilised * 100%/all eggs in concrete variant.

### 2.3. Effect of Inhibitors on Enzymatic Activities

The enzyme inhibitor concentrations were established on the basis of a preliminary experiment, in which the effect of 0.25, 0.5, 1.0, 2.0 and 4 mM of ammonium molybdate (POCh, Gliwice, Poland, acid phosphatase, AcP inhibitor [12]), 0.00625, 0.0125, 0.025, 0.05 and 0.1 mM of gossypol (Sigma-Aldrich, lactate dehydrogenase, LDH inhibitor [13]) and 0.0313, 0.0625, 0.125, 0.25 and 0.5 M of acetamide (Sigma-Aldrich, a water-soluble β-N-acetylglucosaminidase, β-NAGase inhibitor [8]) on the enzymes’ activities in the Eurasian perch seminal plasma and sperm extracts was examined. The semen obtained from the Eurasian perch was subject to centrifugation (8000× *g*, for 10 min) in order to separate the seminal plasma from the spermatozoa. The pellet of the spermatozoa was incubated in 20 mM Tris HCl, with a pH adjusted to 7.6 at room temperature during 30 min and then centrifuged (8000× *g* for 10 min). The seminal plasma and supernatants of the sperm extracts were used to investigate the effect of the enzyme inhibitors on AcP, LDH and β-NAGase activities. Ammonium molybdate and acetamide dissolved in ddH_2_O were added to the seminal plasma and sperm extracts, gossypol was first diluted in ethanol—drop by drop, in the smallest possible volume—and then in ddH_2_O, and the activities of AcP, LDH and β-NAGase were subsequently determined [14,15,16]. The enzymes’ activities were expressed as the percentage of reference activities, the activity of the untreated samples defined as 100%. The seminal plasma and sperm extract without inhibitors were used as the control samples. The concentration of used enzyme inhibitors had to be high enough to decrease the enzyme activities at a visible level.

#### 2.3.1. Determination of the Effect of the Enzyme Inhibitors on Sperm Motility

In the next experiment, once the optimal buffer for the perch gamete activation was established, the collected semen was mixed (1:1) with the Kobayashi immobilising buffer [17] containing 0.5, 2 and 8 mM of ammonium molybdate (AcP inhibitor); 0.0125, 0.05 and 0.2 mM of gossypol (LDH inhibitor); as well as 0.0625, 0.25 and 1 M of acetamide (β-NAGase inhibitor). The final concentrations of the inhibitors were lower by half. Semen mixed with a pure Kobayashi buffer served as the control. Thus, the prepared samples were subjected to computer-assisted semen analysis (CASA). We used an immobilising medium for the fish spermatozoa as the spermatozoa concentration is very high in perch, decreasing their motility during transport to the laboratory as a result. Sperm motility was stimulated in the activation media (80 mM NaCl, 20 mM KCl, 10 mM Tris, pH 8.0 with 0.5% albumin), 15 min after dilution with inhibitor solutions. CASA analysis was undertaken as described above.

#### 2.3.2. Eurasian Perch Fertilisation with Enzyme Inhibitors

**Figure 1:**
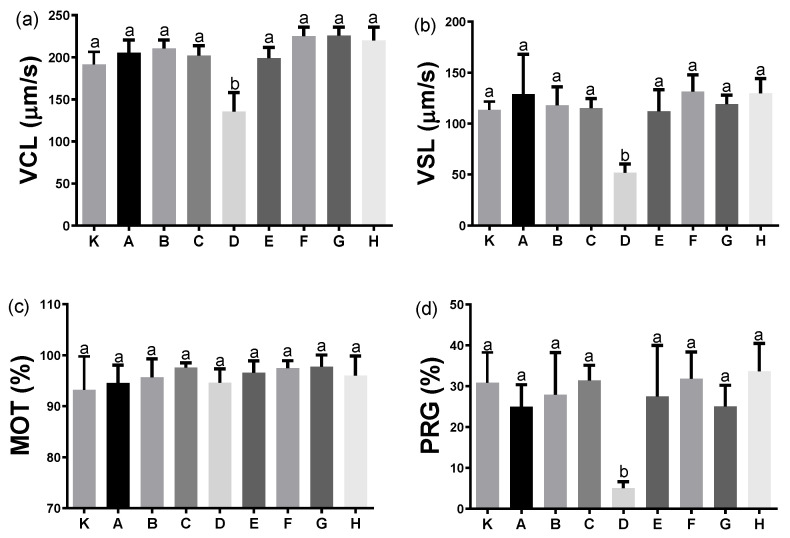
Determination of the influence of different activating buffers on the perch sperm motility parameters: (**a**) VCL, curvilinear velocity of sperm, (**b**) VSL, straight-line velocity, (**c**) MOT, motile sperm and (**d**) PRG, progressive sperm, (K—control, hatchery water; A—„Lahnsteiner” liquid, pH 7.0, 100 mM NaCl, 10 mM Tris; B—„Lahnsteiner” liquid, pH 8.0; C—„Lahnsteiner” liquid, pH 9.0; D—10 mM Tris, 10 mM NaCl, pH 8.5; E—20 mM Tris, 40 mM NaHCO_3_, pH 8.5; F—„Woynarovich” liquid, 0.3% urea, 0.4% NaCl; G—10 mM HEPES, 100 mM NaCl, pH 8.0 and H—80 mM NaCl, 20 mM KCl, 10 mM Tris, pH 8.0). Values are shown as a mean ± SD, *p* ≤ 0.01, *n* = 4. Different letters (a, b) indicate the statistically significant differences between the activating buffers (*p* ≤ 0.01). The data were analysed using a repeated measure one-way analysis of variance (ANOVA), followed by Tukey’s post hoc test.

**Figure 2:**
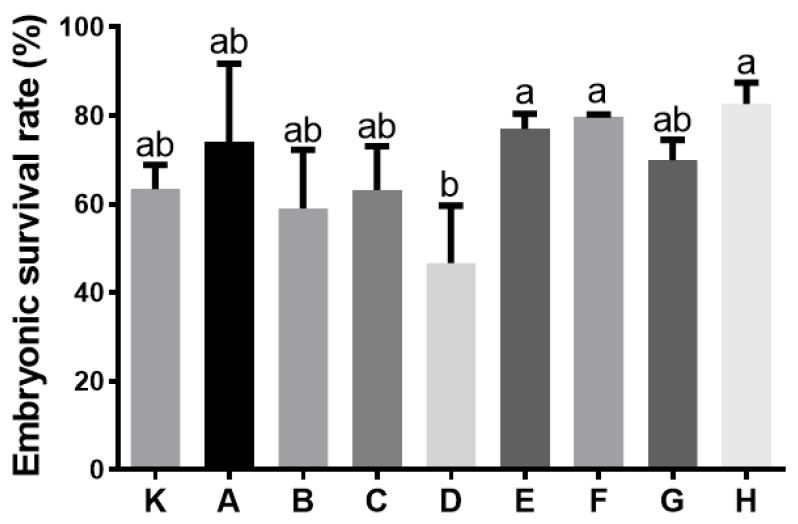
Determination of the influence of different activating buffers on the perch embryonic survival rate (72 h after fertilisation) (K—control, hatchery water; A—„Lahnsteiner” liquid, pH 7.0, 100 mM NaCl, 10 mM Tri; B—„Lahnsteiner” liquid, pH 8.0; C—„Lahnsteiner” liquid, pH 9.0; D—10 mM Tris, 10 mM NaCl, pH 8.5; E—20 mM Tris, 40 mM NaHCO_3_, pH 8.5; F—„Woynarovich” liquid, 0.3% urea, 0.4% NaCl; G—10 mM HEPES, 100 mM NaCl, pH 8.0 and H—80 mM NaCl, 20 mM KCl, 10 mM Tris, pH 8.0). Values are shown as a mean ± SD, *p* ≤ 0.01, *n* = 3. Different letters (a, b) indicate the statistically significant differences between the activating buffers (*p* ≤ 0.01). The data were analysed using a repeated measure one-way analysis of variance (ANOVA), followed by Tukey’s post hoc test.

The effect of the inhibitors of particular enzymes on the fertilisation success was determined, as was described above. In each experimental group, 20 μL of fresh semen was used per 100 eggs; fertilisation was performed in 5 mL of a respective activating buffer. In order to determine the inhibitory effect on perch fertilisation, ammonium molybdate (0.25, 1, 4 mM), buffer osmolality in particular doses of the inhibitor 208, 213, 228 mOsm/kg, respectively, gossypol (0.00625, 0.025, 0.1 mM; osmolality: 211, 228, 306 mOsm/kg) and acetamide (0.03, 0.125, 0.5 M; osmolality: 237, 329, 706 mOsm/kg) were added to the activation medium. The activation medium (the control in this experiment) was composed of 80 mM NaCl, 20 mM KCl and 10 mM Tris, and with a pH of 8.0 (buffer H, see Figure 1 and Figure 2). After 3 min, each dish was rinsed, first with the respective activating medium, to wash out the residues of the spermatozoa, and then with hatchery water. The fertilised eggs were placed in incubators at 14 °C, and after 72 h the embryonic survival rate was determined according to the following formula: eggs that were fertilised * 100%/all eggs in each variant.

All analyses were performed at a significance level of 0.05 using GraphPad Prism 8.0 (GraphPad Software Inc., San Diego, CA, USA). The data were analysed using a repeated measure one-way analysis of variance (ANOVA), followed by Tukey’s post hoc test.

## 3. Results

The Eurasian perch sperm motility parameters were similar with the use of all analysed activation media (Figure 1), except for solution D, which caused a significant decrease in motion parameters, including VCL, VSL and PRG. The percentage of fertilised eggs was highest when solution H was used as the activating buffer (Figure 2). In this case, the standard deviation was also sufficiently small, facilitating the solution’s selection for further experimentation with the aim of determining the effect of adding an enzyme inhibitor on the percentage of fertilised eggs.

Table 2 shows a dose-dependent decrease in AcP, LDH and β-NAGase activities of Eurasian perch seminal plasma and sperm extracts in the presence of ammonium molybdate, gossypol and acetamide, respectively. The lowest doses of particular inhibitors caused at least a 30.1 ± 4.9% inhibition of the enzyme activity, whereas the highest concentrations of inhibitors elicited a decrease in the enzyme activity from 61.2 ± 7.1 to 85.8 ± 8.4%.

**Figure 3:**
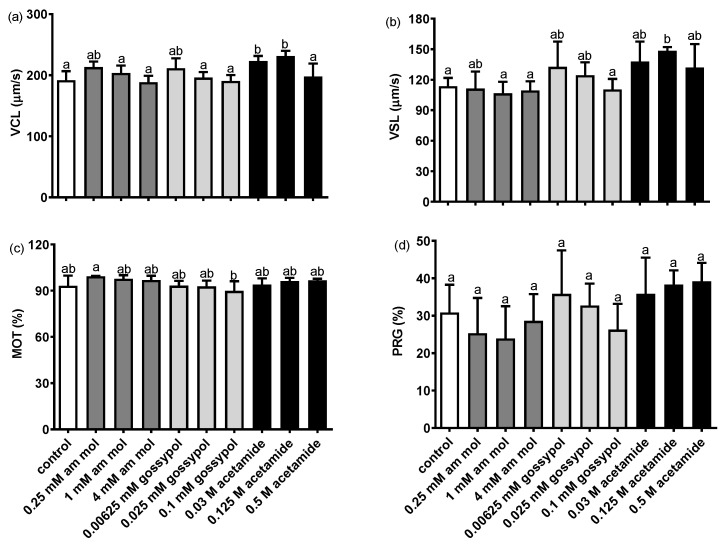
The effect of the enzyme inhibitors added to spermatozoa on the perch sperm motility parameters: (**a**) VCL, curvilinear velocity of sperm, (**b**) VSL, straight-line velocity, (**c**) MOT, motile sperm and (**d**) PRG, progressive sperm. Values are shown as a mean ± SD, *p* ≤ 0.01, *n* = 4. Different letters (a, b) indicate the statistically significant differences between the doses of inhibitors (*p* ≤ 0.01). The data were analysed using a repeated measure one-way analysis of variance (ANOVA), followed by Tukey’s post hoc test.

**Figure 4:**
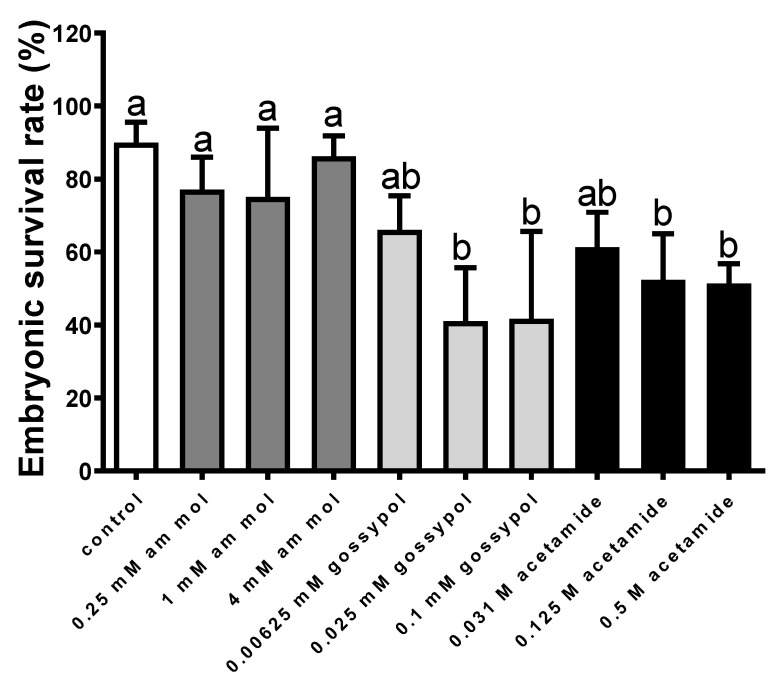
The effect of ammonium molybdate, gossypol and acetamide (inhibitors, respectively, AcP, LDH and β-NAGase) on the embryonic survival rate (72 h after fertilisation). Values are shown as a mean ± S.D, *n* = 3. Different letters (a, b) indicate the statistically significant differences between the doses of inhibitors (*p* ≤ 0.01). The data were analysed using a repeated measure one-way analysis of variance (ANOVA), followed by Tukey’s post hoc test.

The determination of the effect of adding an enzyme inhibitor to the sperm motion parameters demonstrated that none of the applied inhibitors caused their values to decrease (Figure 3). In the case of acetamide, an increase was even observed in the values of the VCL and VSL parameters. The addition of ammonium molybdate caused no significant decrease in the percentage of the fertilised eggs, which, in the sample with 4 mM of molybdate, reached 77.0 ± 8.9%, compared to the 89.9 ± 5.6% noted in the control (Figure 4). The addition of gossypol (except for the lowest dose) reduced the fertilisation rate to 41.1 ± 14.7%. Acetamide was also observed to significantly reduce the percentage of fertilised eggs to 51.4 ± 5.4% (Figure 4).

## 4. Discussion

### 4.1. Determination of the Optimal Activating Solution for Perch Gametes

We sometimes observed the phenomenon of a low fertilisation percentage in practice, despite the fact that everything was well conducted, especially when the fertilisation was in hatchery water. Therefore, it is important to fertilise in buffers of a known composition. The application of CASA for the motility analysis of the perch’s sperm activated with various buffers, demonstrated the greatest effects for buffers E, F and H. However, it is noteworthy that such an outcome was obtained in spite of the differences in the osmolality values of these buffers. Buffer E had the lowest osmolality (101 mOsm/kg) compared to buffers F (181 mOsm/kg) and H (206 mOsm/kg). Despite the fact that perch gametes tolerate such a wide range of osmolality values, the excessively low value has a negative impact on perch gametes, reflected in the decreased values of such sperm motility parameters as VCL, VSL and PRG, and by a lower percentage of fertilised eggs upon activation with buffer D (osmolality of 35 mOsm/kg). This phenomenon is also observed in the cyprinids, as reduced osmolality provides a signal for initiating the motion, but an excessive reduction results in sperm motility disorders [18]. It was already reported that the optimal osmolality of perch sperm activation media ranges between 100 and 200 mOsm/kg [19]. This was confirmed in our study through the analysis not only of sperm motility, but also of the effects of various perch gamete activation media in the egg fertilisation process. It may therefore be concluded that the appropriate value of osmolality is of significance not only for the appropriate activation of sperm motion, but also for the „activation” of oocytes and might be related to the conditions provided by the ovarian fluid in the fertilisation microenvironment.

This study is also the first to compare the effects of so many solutions for perch gamete activation. We demonstrated that by using a specific buffer, it is possible to increase the egg fertilisation rate by as much as 31% (63.4 ± 5.4% in control vs. 82.7 ± 4.7% in buffer H). In addition, that the fertilisation rate was achieved with the use of the Woynarovich solution is consistent with the results reported by Żarski et al. [2], who demonstrated the considerable effectiveness of this solution in the commercial fertilisation of Eurasian perch. Although it causes less success in fertilisation, hatchery water is adequate for spawning in fish culture conditions. Nevertheless, scientific research needs to be conducted with the use of an activating solution with a well-known composition and suitable for the reliable measurement of pH value. Unfortunately, neither water nor the Woynarovich solution are appropriate in this case.

### 4.2. Effect of Inhibitors on Enzymatic Activities, Sperm Motility Parameters and Fertilisation

Enzymes observed in semen might play several roles; for example, AcP in mammals takes part in the conversion of proacrosin to acrosin [20], β-NAGase is associated with the acrosome, and its inhibition in mouse sperm results in a lower percentage of fertilised eggs [21]. Our earlier experiment revealed that the AcP present in ide and sturgeon sperm might have an active role in egg fertilisation [8,9], but we also observed no effect of ammonium molybdate addition during carp fertilisation (Sarosiek, unpublished data). The effect of the inhibitors of individual enzymes was determined using the easily available and widely reported inhibitors of AcP, LDH and β-NAGase [8,12,13]. Unlike gossypol, the inhibitors of AcP and β-NAGase used in this study, i.e., molybdate and acetamide, are non-toxic to fish [22,23]. Ammonium molybdate concentrations that are similar to those applied in our experiments were also reported as inhibiting the AcP activity in Acipenseridae fish [24]. Ciereszko and Dabrowski [13] investigated the effect of gossypol on the LDH activity on the semen of yellow perch (*Perca flavescens*), and achieved 32% inhibition at a gossypol concentration of 100 mM, which is considerably lower than that obtained in our study for Eurasian perch (Table 2). On the other hand, the inhibition of the activity of β-NAGase present in the semen of rainbow trout and Siberian sturgeon required a much higher concentration of acetamide (1 M) than that applied in our study [8]. For this reason, when analysing the effects of enzyme inhibitors from various sources, appropriate doses of inhibitors should always be adjusted to the individual enzymes.

#### 4.2.1. The Effect of Enzyme Inhibitors on Perch Gametes

The addition of AcP, LDH and β-NAGase inhibitors of the immobilising buffer [17] enabled the conclusion that none decreased the values of the sperm motility parameters. The addition of acetamide even increased the values of VCL and VSL, i.e., the parameters strongly associated with successful fertilisation [25]. As demonstrated by other authors, gossypol may decrease the values of sperm motility parameters in perch, but performs this after 2 h of semen incubation with an LDH inhibitor [13]. In our study, the maximum incubation time was 15 min. In spite of the lack of effect of the enzyme inhibitors on the sperm motility parameters, the addition of gossypol and acetamide caused a significant decrease in the fertilisation percentage. Therefore, it is highly likely that the addition of enzyme inhibitors immediately before fertilisation only elicited the inhibition of enzyme activities, with no impact on the sperm motion apparatus. The activities of AcP, LDH and β-NAGase were also reported in both the sperm and oocytes of perch (unpublished data). Most likely, in our experiment, the activity of the enzymes from both sources was inhibited. We suppose that the addition of the enzyme inhibitors during fertilisation did not decrease the oocyte’s potential to be fertilised. Three doses of enzyme inhibitors were used, and between the two highest doses of gossypol and acetamide there were no differences in the embryonic survival rate. If the solution with 0.1 mM gossypol or 0.5 M acetamide were harmful to the oocytes, there would be a statistically lower embryonic survival rate than in the 0.025 mM gossypol and 0.125 M acetamide addition, respectively. Therefore, this fact allows us to suppose that it was the enzyme inhibition effect. Considering our earlier investigations demonstrating how the inhibition of β-NAGase activity in rainbow trout caused a drastic decrease in the percentage of fertilised oocytes [8], the results obtained in the present study for perch seem to confirm the thesis that enzymes play an active role in the fish fertilisation process, in spite of their simplified gamete morphology (the presence of micropyle in eggs, no acrosome in spermatozoa) [26].

## 5. Conclusions

In conclusion, out of the studied solutions for perch gamete activation, the three most effective were buffer E (20 mM Tris, 40 mM NaHCO_3_, pH 8.5), F („Woynarovich” solution 0.3% urea, 0.4% NaCl) and H (80 mM NaCl, 20 mM KCl, 10 mM Tris, pH 8.0). The addition of ammonium molybdate did not cause a significant reduction in the percentage of motile sperm or of the fertilised eggs. The highest dose of gossypol did not reduce the percentage of motile sperm; moreover, the addition of this inhibitor (aside from the lowest dose) caused a decrease in the embryonic survival rate. The addition of acetamide significantly reduced the percentage of embryonic survival rate, but without negatively affecting the sperm motility parameters. The acid phosphatase activity in the perch semen did not affect the process of fertilisation. In contrast, both LDH and β-NAGase seemed to play a very important role in the perch fertilisation process, which needs to be investigated further in the future.

## Figures and Tables

**Table 1:** Composition of the buffers used during the determination of the optimal activating solution for perch sperm motility parameters and fertilisation.

	Composition	pH	Osmolality (mOsm × kg^−1^)
K	Hatchery water	6.9	4
A	100 mM NaCl, 10 mM Tris	7.0	199
B	100 mM NaCl, 10 mM Tris	8.0	198
C	100 mM NaCl, 10 mM Tris	9.0	199
D	10 mM NaCl, 10 mM Tris	8.5	35
E	20 mM Tris, 40 mM NaHCO_3_	8.5	101
F	0.3% urea, 0.4% NaCl	7.7	181
G	10 mM HEPES, 100 mM NaCl	8.0	204
H	80 mM NaCl, 20 mM KCl, 10 mM Tris	8.0	206

**Table 2:** The effect of inhibitors concentration on AcP, LDH and β-NAGase activities as the percentage of the enzyme’s inhibition (%). Values are shown as a mean ± SD, *n* = 3.

	Ammonium Molybdate				
mM	0.25	0.5	1.0	2.0	4.0
Seminal plasma Sperm extract	39.8 ± 5.2 30.1 ± 4.9	46.5 ± 4.8 39.2 ± 5.2	51.6 ± 6.7 58.8 ± 6.1	60.1 ± 7.6 65.3 ± 7.0	85.5 ± 8.4 81.7 ± 6.3
	Gossypol				
mM	0.00625	0.0125	0.025	0.05	0.1
Seminal plasma Sperm extract	37.2 ± 3.9 30.9 ± 3.8	40.5 ± 3.8 37.1 ± 5.5	50.4 ± 5.8 41.3 ± 5.4	55.4 ± 5.9 48.8 ± 5.4	61.2 ± 7.1 64.9 ± 6.9
	Acetamide				
M	0.0313	0.0625	0.125	0.25	0.5
Seminal plasma Sperm extract	31.2 ± 4.0 35.1 ± 5.1	58.3 ± 5.5 44.9 ± 5.9	69.0 ± 7.3 56.3 ± 6.5	73.4 ± 8.1 69.9 ± 7.8	84.6 ± 8.1 77.9 ± 7.9

## Data Availability

The data presented in this study are available on request from the corresponding author. The data are not publicly available for privacy reasons.
